# Evaluating the Effectiveness of a Multimodal Psychotherapy Training Program for Medical Students in China: Protocol for a Randomized Controlled Trial

**DOI:** 10.2196/58037

**Published:** 2025-01-03

**Authors:** Tao Pei, Yinan Ding, Jinsong Tang, Yanhui Liao

**Affiliations:** 1 Mental Health Centre Nanjing Normal University Nanjing China; 2 Department of Psychiatry Sir Run Run Shaw Hospital Zhejiang University School of Medicine Hangzhou China; 3 Department of Psychology and Neuroscience Boston College Chestnut Hill, MA United States

**Keywords:** multimodal teaching, psychotherapy training, Chinese medical students, randomized controlled trial

## Abstract

**Background:**

Psychotherapy is central to the treatment of mental disorders, highlighting the importance of medical students and residents developing competencies in this area. Chinese medical residents have expressed a strong need for psychotherapy training, yet they are generally dissatisfied with the current offerings. This paper presents the protocol for an evidence-based, well-structured psychotherapy teaching program aimed at medical students and residents.

**Objective:**

This study involves a randomized controlled trial of a 2-day multimodal intensive educational intervention designed to evaluate the effectiveness of a new psychotherapy teaching program for medical students and residents in China. The primary outcomes include participants’ knowledge and utilization of psychotherapy, training program acceptability, self-reported self-efficacy, and motivation to apply psychotherapy.

**Methods:**

This 2-arm randomized controlled trial was conducted at Sir Run Run Shaw Hospital. The study aimed to recruit approximately 160 medical students and residents, with about 80 participants in the intervention group and 80 in the control group. Both groups completed a baseline survey before participation, reporting their psychotherapy knowledge, utilization of psychotherapy, self-efficacy, and self-motivation. The intervention group received a 2-day multimodal intensive educational intervention (supervision-based online teaching), while the waitlist control group did not receive any intervention during this period. Both groups were followed up for 8 weeks, completing the same survey administered at baseline. At the end of the study, the control group received the intervention. The primary outcome measure was the change in trainees’ psychotherapy knowledge before and after the intervention training. Secondary outcome measures included changes in the trainees’ utilization of psychotherapy, self-reported self-efficacy, and self-reported motivation for psychotherapy. Additionally, training program acceptability was assessed. Analysis of covariance was used to analyze the primary outcomes. Pearson correlations and regression analysis explored factors associated with the knowledge score at baseline. The secondary outcomes, including participants’ psychotherapy utilization, confidence, and motivation, were analyzed using the same methods as for knowledge. All tests were 2-tailed, with a significance level set at *P*<.05.

**Results:**

A total of 160 participants were recruited and randomized between January 4 and 12, 2024. Baseline assessments were conducted from January 28 to February 1, 2024. The psychotherapy training program for the intervention group took place on February 3 and 4, 2024. Posttraining assessments were conducted starting April 1, 2024. Due to withdrawals, incomplete surveys, and data loss, we had a total of 113 participants: 57 in the intervention group and 56 in the control group. The amount of data varied across measures. The data analysis was finished in August 2024.

**Conclusions:**

This study aims to evaluate the effectiveness of the multimodal psychotherapy training program for medical students in China. If this brief, cognitive behavioral therapy–based psychotherapy skill training proves effective, the potential mental health impact of its nationwide expansion could be significant.

**Trial Registration:**

ClinicalTrials.gov NCT06258460; https://clinicaltrials.gov/ct2/show/NCT06258460

**International Registered Report Identifier (IRRID):**

DERR1-10.2196/58037

## Introduction

### Background

The delivery of psychosocial and psychotherapeutic interventions remains central to the treatment of many patients with psychiatric disorders (eg, obsessive-compulsive disorder, panic disorder, major depression, eating disorders, and addictive behaviors) and psychosomatic disorders (eg, hypertension, bronchial asthma, and rheumatoid arthritis) [1,2]. Cuijpers et al [3] conducted a large-scale network meta-analysis to examine the effects of various types of psychotherapies for adult depression, including cognitive behavioral therapy (CBT), interpersonal therapy, psychodynamic therapy, problem-solving therapy, behavioral activation, life-review therapy, and “third-wave” therapies, as well as nondirective supportive counseling. They found that all types of these therapies were more effective than care-as-usual and waiting list control conditions and that most therapies were more efficacious than placebo. Additionally, most therapies maintained significant effects at the 12-month follow-up compared with care-as-usual. A strong evidence base indicated that several psychotherapy modalities were effective for most mental disorders, whether used alone or in combination, primarily including behavior therapy, CBT, and interpersonal psychotherapy [4,5]. According to a systematic review and meta-analysis, the emotional change processes and mechanisms of psychotherapy were most strongly associated with specific CBT methods, such as fear habituation, emotion regulation and experience, and the habitual reorganization of maladaptive emotional perceptions [6]. Today, CBT is recommended as a first-line intervention for both the acute treatment and relapse prevention of various mental illnesses, including major depressive disorder, and most patients prefer psychological treatment over pharmacologic options [5]. Therefore, medical students in clinical rotations and residents working in psychiatric and psychosomatic departments should be required to develop competencies in psychotherapy, particularly in CBT [7]. However, there remains a significant treatment gap for mental disorders in China [8]. Despite the high prevalence of mental disorders in the country, medical professionals in psychiatric or mental health departments often have a comparatively low capacity to provide adequate care and lack qualified training in psychotherapy. Learning basic psychotherapy skills would benefit medical students, residents, and other health care providers (HCPs), including doctors and nurses, across all departments. These skills can be applied in clinical practice to enhance doctor-patient relationships [9]. A study conducted in Beijing found that after participating in a 2-year psychotherapy training program, medical doctors reported improvements in diagnosing and treating mental illness, as well as in doctor-patient communication and the development of strong doctor-patient relationships. Patients also demonstrated significant improvements in levels of depression and anxiety, the severity of physical symptoms, quality of life, and the patient-rated therapeutic relationship [9]. Therefore, psychotherapy training for medical students, residents, and other HCPs requires greater emphasis.

A narrative review indicated that medical residents in psychiatry have a strong need for psychotherapy training to enhance their competence, yet they generally express dissatisfaction with the current training programs [10]. Therefore, providing evidence-based and well-designed psychotherapy training programs is essential for equipping medical students and residents with a foundational understanding of psychotherapy and the necessary skills for clinical practice [11]. Compared with traditional methods, psychotherapy teaching that emphasizes skill practice and role experience is more popular and effective. For instance, a single-day simulation training program has proven effective in psychiatry for medical students, enhancing their knowledge, communication and interview skills with patients, and confidence in treatment [12]. Despite the critical importance of learning psychotherapy skills during medical school and residency, there is a notable lack of formal evaluation of psychotherapy teaching methods and their efficacy. Given the lack of access to psychotherapy, medical and psychological educators, along with program directors, should design high-quality curricula to teach medical students, residents, and other HCPs the essential knowledge and skills in psychotherapy. These curricula should be evaluated through well-conducted, methodologically robust randomized controlled trials (RCTs) [13].

### Aim and Hypotheses

To evaluate the effectiveness of the multimodal psychotherapy training program for medical students in China, well-designed RCTs of psychotherapy teaching programs are urgently needed. The primary aim of this proposed project is to assess the effectiveness of a new multimodal psychotherapy teaching program for medical students and residents in China, which is designed to enhance their psychotherapeutic skills and improve their performance in entry-level clinical settings. The primary hypothesis was that, compared with a control intervention, the intervention group receiving the psychotherapy teaching program would acquire significantly more knowledge about psychotherapy after training. We also hypothesized that the program would lead to an increase in the utilization of psychotherapy and be associated with improved knowledge in this area. The third hypothesis posited that trainees’ self-reported self-efficacy and motivation to apply psychotherapy in clinical practice would increase significantly.

## Methods

### Patient and Public Involvement

Neither participants nor the public were involved in the trial’s design, recruitment, or conduct of this study.

### Study Design and Participants

**Figure 1:**
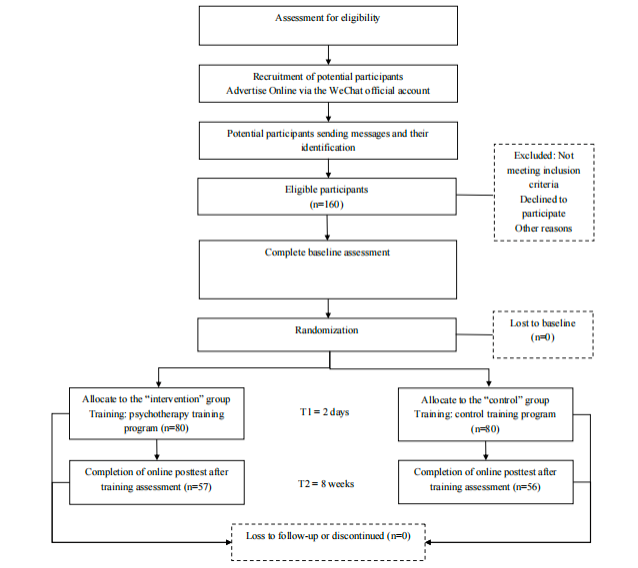
Flowchart study design.

This study was an RCT of a 2-day multimodal intensive educational intervention aimed at enhancing the clinical skills in psychotherapy of Chinese medical students and residents. The trial included a waitlist control group, with 8 weeks of follow-up for all participants. A detailed schedule of the study procedures is summarized in Figure 1.

Study participants (n=160) primarily included medical students, residents, and a few other HCPs, such as doctors and nurses, in China. There were no specific restrictions for participants, although most were from Zhejiang University School of Medicine. An overview of participant eligibility criteria is provided in Textbox 1.

**Study inclusion criteria (participants who did not qualify for the criteria were not recruited to participate in this study).**

- Medical students, residents, and other health care providers
- Aged 18 years or older
- Expressing an interest in psychotherapy
- Willing to receive randomization
- Willing to provide informed consent to participate in the study

### Sample Size and Power Calculation

The sample size was calculated based on the primary outcome using G*Power [14] (version 3; Universität Düsseldorf). As the data were continuous variables and analysis of covariance (ANCOVA) would be used to compare differences between the 2 groups, an a priori analysis selecting ANCOVA as the statistical test was conducted to determine the required sample size. As only a few studies have examined the effectiveness of psychotherapy training for medical students, we were unable to reference effect size or other data as criteria. Therefore, a medium effect size for the primary outcome was estimated preliminarily. It was determined that 128 participants (64 in each group) would be required to achieve 80% power (effect size *f*=0.25; 1 – β=0.80; α=.05). To account for potential attrition, we aimed to recruit approximately 160 participants (80 in each group), ensuring that the proposed analysis would be sufficiently powered even if 25% of participants in each group were lost to attrition.

### Randomization and Group Allocation

This study aimed to recruit approximately 160 participants, with about 80 in each group. The study coordinator randomized participants into the intervention and control groups using a random number generator in R software, maintaining a 1:1 ratio for allocation to the experimental and control conditions. Participants in the intervention group first received the experimental condition, which included the psychotherapy training program after the baseline assessment. The waitlist control group received the control condition, meaning they participated in the psychotherapy training program only at the end of the study. The timeline of the study was as follows: all participants underwent a baseline assessment, the intervention group received the training program, and the control group received a control training program. After 8 weeks, all participants were followed up, and finally, the control group received the intervention training program (Figure 1).

### Recruitment

The researchers advertised the program online through the WeChat (Tencent Holdings Limited) official account of the Department of Psychiatry at Sir Run Run Shaw Hospital, Zhejiang University School of Medicine, to recruit potential participants. They encouraged sharing the program details with medical schools and hospitals. Interested individuals could register by sending messages and their identification to the research assistants (Zitang Zhou and Luyao Zou). Research assistants then contacted the respondents to assess their eligibility, explain the study to each participant, and inform them about the allocation to either the control or intervention groups, where they would receive the psychotherapy training program.

### Baseline and Posttraining Data Collection

Before randomization, demographic information and self-reported questionnaires were collected from all participants at baseline. This information included participants’ sex, age, identity (student, resident, or HCP), education level (undergraduate or graduate degree), department affiliation (psychiatric or nonpsychiatric), and years of psychotherapy-related work. Additionally, outcome measurements were gathered, including trainees’ knowledge of psychotherapy, utilization of psychotherapy, self-reported self-efficacy, and self-reported motivation, among others. The questionnaires on psychotherapy knowledge, utilization, and self-efficacy and motivation to engage in psychotherapy were specifically designed based on our training program. Outcome measurements were assessed again 8 weeks after training (Table 1). Data were collected online using WenJuanXing (Questionnaire Star), a Chinese platform that provides professional online questionnaire surveys and data collection for RCTs [15]. The hospital’s data monitoring committee oversaw the data collection process, and personal information was deidentified to ensure confidentiality.

**Table 1:** Schedule of enrollment and posttraining assessments.^a^

Schedule	Baseline	8 weeks after training
Initial approach	✓	N/A^b^
Informed consent	✓	N/A
Eligibility screen	✓	N/A
Randomization	✓	N/A
Intervention/control initiation	✓	N/A
Demographic characteristics	✓	N/A
Knowledge	✓	✓
Self-reported self-efficacy	✓	✓
Self-reported motivation	✓	✓
Utilization	✓	✓

^a^This table illustrates the schedule of enrollment and posttraining assessments. Initial approach, informed consent, eligibility screen, randomization, intervention/control initiation, and demographic characteristics were evaluated and collected only at the baseline timeline. Psychotherapy knowledge, self-reported self-efficacy, self-reported motivation, and utilization were both collected at the baseline and 8 weeks after the training.

^b^N/A: not applicable.

### Development of the Psychotherapy Training Program

The psychotherapy training program was primarily developed by an experienced psychotherapist (Tao Pei) and an MD-level psychiatrist (Yanhui Liao), both of whom have approximately 20 years of relevant experience. The details of the 2-day psychotherapy training program are outlined in Multimedia Appendix 1. The program included 2 days of intensive training followed by 8 weeks of follow-up, with guidance on applying psychotherapy in clinical settings.

### Intervention

#### Control Group

After providing consent, participants allocated to the waitlist control group received a message encouraging them to complete all questionnaires from baseline through to the final follow-up at 8 weeks. They were sent messages via WeChat to thank them for their participation and to remind them of the timeline for completing the study. Once they finished the posttraining measurement, a digital booklet of the psychotherapy training program was provided to them via WeChat or as a hard copy upon request. After the trial concluded, participants in the control group were offered the opportunity to receive the psychotherapy training program free of charge.

#### Intervention Group

All participants in the intervention group received the 2-day psychotherapy training program and were given a hard copy of the program booklet at recruitment. Additionally, supervision-based group meetings were held during the follow-ups at weeks 1, 2, 4, and 8, with each meeting lasting approximately 2 hours. During these follow-up meetings, instructors—including psychotherapists and psychiatrists—were available to answer any psychotherapy-related questions, encourage participants to practice psychological interventions, and provide further information to support the clinical application of psychotherapy.

### Outcomes and Outcome Measures

#### Primary Outcome

Trainees’ psychotherapy knowledge was assessed by participants using an 11-point scale (ranging from 0 to 10) before and after the 8-week period (Table 2). At baseline, 160 participants completed the self-reported psychotherapy knowledge questionnaire, and the results indicated a high internal consistency coefficient (Cronbach α) of 0.980 for the scores across the 17 items.

**Table 2:** Knowledge about psychotherapy.^a^

Variables	Measures (0-10)
Overview of psychotherapy	
Supportive psychotherapy techniques	
Overview of cognitive behavioral therapy	
Beck’s cognitive therapy	
Identify automatic thinking and do cognitive conceptualization	
Cognitive conceptualization	
Evaluation of automatic thinking	
Reconstruction techniques for automated thinking1: Socratic questioning	
Reconstruction techniques for automated thinking2: pie charts, continuous spectrum, cost-benefit analysis, behavioral experiments	
Challenge automatic thinking	
Social skill training	
Problem-solving	
Behavioral therapy theory and behavioral conceptualization	
Behavioral activation	
Relaxation training	
Exposure therapy	
Competency structure of psychotherapists and the growth path of cognitive behavioral therapy therapists	

^a^This is the assessment of participants’ knowledge about psychotherapy. Each item was rated on a 0-10 scale, where 10 means “knowing very well.”

#### Secondary Outcomes

##### Training Program Acceptability

Program acceptability in the intervention group was measured using questions designed to assess acceptability, as detailed in Table 3.

**Table 3:** Questions for assessing the psychotherapy training program acceptability.^a^

Category and questions		Rating
**General**		
	Overall rating of the program	5=like very much; 4=like somewhat; 3=neutral; 2=dislike somewhat; 1=very dislike; 5=very likely
**Appraisal**		
	Appraisal of the program—the likelihood of applying the program for patients Appraisal of the program—the likelihood of recommending the program to other medical students or other health care providers	5=very likely; 4=somewhat likely; 3=neutral; 2=unlikely; 1=not at all likely
**Acceptability**		
	I would have been able to help patients to deal with mental problems with the program The program made it easier to communicate and help patients during clinical work The program disrupted my daily schedule The program is easy to understand	5=strongly agree; 4=agree; 3=neutral; 2=disagree; 1=strongly disagree
**Frequency**		
	Frequency of using psychotherapy	1=almost never; 2=sometimes; 3=always

^a^This is the rating of the training acceptability assessment. The last item, “Frequency of using psychotherapy,” was rated on a 1-3 Likert scale, where 3 indicates “always.”

##### Utilization of Psychotherapy

The utilization rate of psychotherapy interventions for patients during the 8 weeks of follow-up was assessed using items from Table 4.

**Table 4:** The utilization rate of psychotherapy.^a^

Variables	Measures (0-10)
Supportive psychotherapy techniques	
Social skill	
Problem-solving skill	
Behavioral activation	
Relaxation training	
Exposure therapy	

^a^This is the assessment of participants’ utilization rate of psychotherapy. Each item was rated on a 0-10 scale, where 10 means maximum utilization and 0 means minimum utilization.

##### Self-Reported Self-Efficacy and Self-Reported Motivation

Self-efficacy and motivation were measured using a visual analog scale on a 10-cm line, representing a continuum from “no self-efficacy or motivation” to “the strongest self-efficacy or motivation” (Table 5).

**Table 5:** Self-reported self-efficacy and motivation of psychotherapy.^a^

Variables		Visual analog scale
**Self-reported self-efficacy**		
	Confidence in practicing psychotherapy	
	Confidence in practicing supportive psychotherapy	
	Confidence in practicing cognitive behavioral therapy	
**Self-reported** **m** **otivation**		
	Willingness or motivation to practice psychotherapy	
	Willingness or motivation to practice supportive psychotherapy	
	Willingness or motivation to practice cognitive behavioral therapy	

^a^This is the assessment of participants’ self-reported self-efficacy and self-reported motivation. Each item was measured by the visual analog scale on a 10-cm line that represents a continuum between “no self-efficacy or motivation” and “the strongest self-efficacy or motivation.

All secondary outcomes were reported as mean values. According to the baseline measurements of our study, 160 participants evaluated their psychotherapy utilization, confidence, and motivation using self-reported questionnaires. The internal consistency coefficients (Cronbach α) for these 3 questionnaires were 0.950, 0.961, and 0.936, respectively.

### Procedures

Figure 1 and Table 1 summarize the schedule for enrollment and posttraining assessments. Participants were evaluated at baseline (0 weeks), before and after receiving the intervention training, and at 8 weeks after training. They were also asked to maintain a daily log of their utilization of the psychotherapy training program, which included activities such as providing emotional support and teaching patients breathing techniques for stress relief. Reminders were sent to participants who did not complete the 8-week posttraining questionnaires.

### Withdrawal From the Program

All participants were free to withdraw from the trial at any time without needing to provide a reason. Following the intention-to-treat principle [15], participants who did not respond to the 8-week posttraining assessment were retained in the analysis according to the group to which they were randomized, regardless of whether they received the intervention. Participants who withdrew were excluded from the analysis, and their reasons for withdrawal were recorded. A complete case analysis was conducted, excluding any participants who withdrew at the posttraining assessment point.

### Data Analysis

All data were automatically collected via WenJuanXing through a WeChat-based link. The data were downloaded from the WenJuanXing database into a user-specific Excel file. This study did not include interim analyses; data were analyzed only after all had been collected. The trial statistician blinded the intervention assignment in the data using R software (R Foundation for Statistical Computing) and SPSS (2013 release; IBM Corp.).

The primary hypothesis was that, compared with the control intervention, the intervention group receiving the psychotherapy teaching program would significantly gain more knowledge about psychotherapy after training. We also hypothesized that the program would lead to increased utilization of psychotherapy and be associated with improvements in knowledge. The third hypothesis posited that trainees’ self-reported self-efficacy and motivation to apply psychotherapy in clinical practice would increase significantly.

Descriptive statistics were used to assess demographic and psychotherapy-related characteristics at baseline. Following normality and homogeneity of variance tests, ANCOVA was used to analyze the primary outcomes. In the ANCOVA model, the dependent variable was the change in the knowledge score. The fixed factor was the group (intervention group or control group), while the covariates included the mean score of psychotherapy knowledge at baseline and years of psychotherapy experience. Pearson correlations and regression analysis (both linear and binary regression models) were used to explore factors associated with the knowledge score at baseline and to assess the increase in psychotherapy utilization in both the intervention and control groups. The secondary outcomes—participants’ psychotherapy utilization, confidence, and motivation—will also be analyzed in the same manner as knowledge.

A complete case analysis was performed, excluding any participants with missing information on the posttraining assessment. Additionally, a sensitivity analysis of the missing data was conducted to determine whether the missingness is random. Demographic information and scores for psychotherapy knowledge, utilization, confidence, and motivation at baseline were compared between the complete data group and the missing data group. All tests were 2-tailed, with a significance level set at *P*<.05.

### Safety and Adverse Events

Throughout the psychotherapy training program, adverse events were closely monitored. Participants were encouraged to communicate any psychotherapy-related issues or adverse events encountered during their clinical work. We prompted each participant to report any adverse events experienced at each group meeting, and they could also report them at any time. If participants experienced severe adverse events, they were encouraged to seek support from a psychologist or psychiatrist.

We did not anticipate any training-related serious adverse events (SAEs), such as life-threatening incidents, during this trial. However, if any adverse events occur, we will document the SAE, record it on the SAE form, and submit it to the ethics committee of Sir Run Run Shaw Hospital (the principal investigator’s affiliation) within 24 hours.

### Ethics Approval and Consent to Participate

This study was approved by the ethics committee of Sir Run Run Shaw Hospital, an affiliate of Zhejiang University School of Medicine (2024 Ethics Approval File No. 2024-0066). The trial was conducted in accordance with the Declaration of Helsinki. Participants were provided with informed consent before the baseline assessment. After thoroughly reading and understanding the content of the consent, they received a link to electronically sign their name at the end of the informed consent form and submit it via WeChat. Each participant was informed about the study’s purpose, procedures, measurements, potential risks, and benefits before recruitment. Informed consent was then obtained from each participant. Participation was entirely voluntary, and participants could withdraw from the study at any time. Coordinating researchers’ contact information was provided to all participants for any inquiries or concerns.

### Data Security

The authors utilized the professional version of WenJuanXing, which features high-level security management, alongside an applet on WeChat that ensures the secure and confidential protection of participants’ data.

## Results

This study recruited 160 participants from January 4 to January 12, 2024. The 2-day training program took place on February 3 and February 4, 2024, and the posttraining assessment was completed on April 1, 2024. Due to withdrawals, incomplete surveys, and data loss, we had a total of 113 participants: 57 in the intervention group and 56 in the control group. The amount of data varies for each measure. Data analysis was completed in August 2024. The results will be published in peer-reviewed journals. If found to be effective, the psychotherapy training program and the accompanying program booklet will be made freely available to the public by the end of the trial.

## Discussion

To our knowledge, this will be the first RCT to evaluate the efficacy of a multimodal psychotherapy training program for medical students in China.

The strength of this study lies in its theoretical framework, primarily guided by cognitive behavioral theory. With a large sample size, this RCT evaluates the efficacy of the psychotherapy training program using multimodal teaching methods in China. If effective, this multimodal psychotherapy training program could be applied nationwide, significantly enhancing its potential impact on public health. Its expansion could help HCPs acquire the necessary psychotherapy skills to effectively manage patients’ psychological issues.

There are several limitations to this study. First, the effectiveness of teaching and the quality of learning can be influenced by various factors, such as opportunities to implement psychotherapy practice, the intensity of clinical work during the follow-up period (including the impact of holidays), and the availability of continuing education resources. These factors cannot be adequately controlled in this study. Second, the 2-day training will take place at Sir Run Run Shaw Hospital, which may deter participants from other regions of China from attending the program. Third, there are only 2 main instructors (Psychiatrist YL and Psychologist TP) involved in this training program. While both have nearly 20 years of teaching and clinical experience, their individual teaching styles and characteristics may still influence the overall effectiveness of the program. Fourth, although this 8-week training program aims to enhance the acquisition of therapeutic skills, developing proficiency in psychotherapy is likely to be a more prolonged process. Fifth, all measures rely on participants’ subjective assessments, which may be influenced by personal biases and expectations of improvement following the intervention training. Lastly, we submitted this protocol to the journal during the recruitment process.

In conclusion, this is the first RCT to evaluate the efficacy of a multimodal psychotherapy training program for medical students in China. If this educational program, which offers brief and short-term psychotherapy skills training, proves effective, its nationwide expansion could have a significant health impact. It provides evidence-based psychotherapy training—primarily in CBT—for medical students, and its dissemination will equip HCPs to better manage mental health issues, such as stress and depression. Therefore, it is crucial to develop effective psychotherapy training that emphasizes basic psychotherapy skills. The results of this training’s effectiveness can offer valuable insights for the future development of training programs, enabling them to better meet the learning needs of medical professionals and enhance doctor-patient relationships.

## Appendix

Multimedia Appendix 1 The details of the 2-day psychotherapy training program.

## Abbreviations

ANCOVA: analysis of covariance
CBT: cognitive behavioral therapy
HCP: health care provider
RCT: randomized controlled trial
SAE: serious adverse event

## Availability of Data and Materials

Data sets from this study and the corresponding analysis code will be made publicly available at the conclusion of the study’s analyses.
