# Correction: Evaluating the Effectiveness of a Multimodal Psychotherapy Training Program for Medical Students in China: Protocol for a Randomized Controlled Trial

**DOI:** 10.2196/71220

**Published:** 2025-02-05

**Authors:** Tao Pei, Yinan Ding, Jinsong Tang, Yanhui Liao

**Affiliations:** 1 Mental Health Centre Nanjing Normal University Nanjing China; 2 Department of Psychiatry Sir Run Run Shaw Hospital Zhejiang University School of Medicine Hangzhou China; 3 Department of Psychology and Neuroscience Boston College Chestnut Hill, MA United States

In “Evaluating the Effectiveness of a Multimodal Psychotherapy Training Program for Medical Students in China: Protocol for a Randomized Controlled Trial” ([JMIR Res Protoc 2025 Jan 3:14:e58037.]doi:10.2196/58037) the authors noted one error.

In “Table 3,” the “Rating” for “Appraisal” appeared as follows:

“4=somewhat likely; 3=neutral; 2=unlikely; 1=not at all likely”

This has been corrected as follows:

"5=very likely; 4=somewhat likely; 3=neutral; 2=unlikely; 1=not at all likely"

The correction will appear in the online version of the paper on the JMIR Publications website on 05 Feburary 2025, together with the publication of this correction notice. Because this was made after submission to PubMed, PubMed Central, and other full-text repositories, the corrected article has also been resubmitted to those repositories.

