# Supplementary material for: Evaluating the Effectiveness of a Multimodal Psychotherapy Training Program for Medical Students in China: Protocol for a Randomized Controlled Trial
Source: JMIR Res Protoc. 2025 Jan 3;14:e58037. doi: 10.2196/58037 (PMC11748421; doi:10.2196/58037)
Supplement: Multimedia Appendix 1 [file resprot_v14i1e58037_app1.docx]

| **DAY 1** | | | | | |
| --- | --- | --- | --- | --- | --- |
| **Time** | **Structure** | **Title** | **Main contents** | **Description and notes** | **Duration** |
| 8:00-8:05 | Leading-in | Introduction | Welcome, greetings, and introduction | Sign an informed consent form for ethics and group rules in advance.  Short greetings and welcome, self-introduction for the teacher, and explaining the content, format, objectives, and requirements of the course. | 5 min |
| 8:05-8:20 | Module 1  Supportive Psychotherapy | Q1 | What clinical contexts can supportive psychotherapy be used in?  What techniques does supportive psychotherapy require to master? | Divide participants into 10 small groups in advance and discuss within groups, paying attention to recording.  Make self-introductions in group to familiarize with each other.  Choose a representative each time to record the discussion processes and results.  Make each group have equal opportunities to speak.  Group 1 to 5 discuss Question 1, and Group 1 answers  Group 6 to 10 discuss Question 2, and Group 6 answers | 15 min |
| 8:20-8:40 |  | Lecture 1 | Overview of psychotherapy and supportive psychotherapy techniques | The importance and irreplaceability of psychotherapy.  Highlighting the importance of therapeutic relationships, the characteristic and operational components of therapeutic relationships.  Select some participatory technologies to highlight their supportive role. | 20 min |
| 8:40-9:15 |  | Demonstration 1 | Supportive psychotherapy for a case of depression | The teacher and assistant play the roles of therapist and patient respectively.  Prepare a case of mild-to-moderate depression.  Introduce main information of the case before the demonstration and remind students to pay attention to what techniques and content they have seen.  Practice establishing therapeutic relationships and providing support during collecting information.  Give feedback and ask questions after the demonstration. | 35 min |
| 9:15-9:50 |  | Exercise 1 | Supportive Psychotherapy | Role playing settings.  Students work in pairs, playing the roles of therapist and patient respectively.  Aim at exercising techniques rather than solving the problems of the case.  Choose cases with moderate rather than high difficulty and try not to use oneself as a case.  Personality disorders are not going to be options.  Give feedback in two-person group practice after the demonstration.  Small groups feedback and questioning. | 35 min |
| 9:50-10:05 | Coffee Break | | | | 15 min |
| 10:05-10:20 | Module2  Overview of CBT and Beck's Cognitive Therapy | Q2 | What kind of psychotherapy do we need?  How to deal with cognition problems in the occurrence and development of mental disorders? | Group 1-5 discuss question 2, and Group 2 answers.  Group 6-10 discuss question 1, and Group 7 answers. | 15 min |
| 10:20-11:00 |  | Lecture 2 | Overview of CBT | Introduction: limitations of only providing supportive treatment.  Introduce the development background of CBT (horizontal and vertical).  Definition of CBT and its core elements.  Wide range of indications, definite therapeutic effects, and specialized disease manuals.  History and development of CBT.  Five advantages and ten characteristics. | 20 min |
|  |  |  | Beck's Cognitive Therapy | In basic concepts, emphasize the explanation of automatic thinking.  Introduce the horizontal model.  Highlighting the core role of automatic thinking in symptom changes.  How to make treatment structured? | 20 min |
| 11:00-11:30 |  | Demonstration 2 | Leading in CBT | Use the case in Demonstration section.  Based on the specific situation of the case，explain the basic theoretical concepts of CBT, introduce the treatment methods and overview, then demonstrate how to do psychoeducation, and infuse hope. | 30 min |
| 11:30-12:00 |  | Exercise 2 | Leading in CBT | Choose depression and adaptive disorders. | 30 min |
| 12:00-13:30 | Lunch Break | | | | 90 min |
| 13:30-13:45 | Module3  Identify automatic thinking and do cognitive conceptualization | Q3 | What cognitive content do different patients have?  Why do different patients have different cognition? | Group 1-5 discuss question 1, and Group 3 answers.  Group 6-10 discuss question 2, and Group 8 answers. | 15 min |
| 13:45-14:05 |  | Lecture 3 | Identify automatic thinking and do cognitive conceptualization | Distinguish between automatic thinking, situation, and emotion.  Common methods for identifying automatic thinking.  Arrow down technique for identifying beliefs.  Demonstration of cognitive conceptualization diagram.  Use DTR tables to record automatic thinking. | 20 min |
| 14:05-14:35 |  | Demonstration 3 | Identify automatic thinking and do cognitive conceptualization | Take GAD as a case.  Lock in specific situations, confirm thoughts and emotional responses at the time, and score.  Identify beliefs, trace early experiences, form preliminary conceptualization and share.  Assign DTR homework. | 30 min |
| 14:35-15:05 |  | Exercise 3 | Cognitive conceptualization | Do not choose cases with fewer cognitive symptoms. | 30 min |
| 15:05-15:20 | Coffee break | | | | 15 min |
| 15:20-15:35 | Module4  Evaluation and Reconstruction of Automatic Thinking | Q4 | What are the typical characteristics of patient cognition?  What are the methods to change cognition? | Group 1-5 discuss question 2, and Group 4 answers.  Group 6-10 discuss question 1, and Group 9 answers. | 15 min |
| 15:35-15:55 |  | Lecture 4 | Evaluation of automatic thinking | Treatment sequence and basis.  Present common types of cognitive distortions, giving examples.  Invite students to share common examples of cognitive distortions. | 10 min |
|  |  |  | Reconstruction techniques for automated thinking1：  Socratic questioning | Guided discovery, and Socratic questioning.  Positive and negative evidence method, role-play. | 10 min |
| 15:55-16:10 |  | Demonstration 4 | Socratic questioning | Present an anxiety case with the main complaint of "my illness cannot be cured."  Use positive and negative evidence and role-playing to challenge automatic thinking. | 15 min |
| 16:10-16:25 |  | Lecture5 | Reconstruction techniques for automated thinking2：  Pie charts, continuous spectrum, cost-benefit analysis, behavioral experiments | Pie charts, continuous spectrum, cost-benefit analysis, behavioral experiments.  Indications and usage, operating procedures, combine case into explanation. | 15 min |
| 16:25-16:50 |  | Demonstration 5 | Pie charts, continuous spectrum, cost-benefit analysis, behavioral experiments | Self-accusation with the expression of "It's all my fault that the kid failed the exam."  Depression with the expression of "I am a failed person."  Eating disorders with the expression of "Should I go on a diet?"  Body image disorder with the expression of "Others will think I look strange." | 25 min |
| 16:50-17:20 |  | Exercise 4 | Challenge automatic thinking | Pick the right automatic thought.  Use a variety of techniques to try to challenge and replace negative automatic thinking. | 30 min |
| 17:20-17:30 |  | Lecture 6 | End the treatment | When and how beliefs change.  Preparation for the end of treatment.  Relapse prevention and balanced life. | 10 min |
| 18:30-19:30 | Bonus | Evening lecture | Social skill training | Indications and usage, design content, operation method, take social anxiety as an example. | 60 min |
|  |  |  | Problem Solving | Indications and usage, operating procedures.  Take the convalescent schizophrenics as an example. |  |
| DAY 2 | | | | | |
| **Time** | **Structure** | **Title** | **Main contents** | **Description and notes** | **Duration** |
| 8:00-8:15 | Module5  Behavioral theory and micro functional behavioral analysis | Q5 | What is the role of behavior in the occurrence and development of mental disorders?  What are the different behavioral symptoms that different patients may have?  What kinds of factors will affect behavior? | Groups 1-3 discuss question 1, and Group 1 answers.  Groups 4-7 discuss question 2, and Group 5 answers.  Groups 8-10 discuss question 3, and Group 10 answers. | 15 min |
| 8:15-8:45 |  | Lecture 7 | Behavioral therapy theory and behavioral conceptualization | Overview of behavioral therapy.  Three behavioral theories.  Type of problem behavior.  SRC model and corresponding technology of each part. | 30 min |
| 8:45-9:15 |  | Demonstration 6 | SRC | Take phobias as an example | 30 min |
| 9:15-9:45 |  | Exercise 5 | SRC | Select cases with prominent behavioral symptoms. | 30 min |
| 9:45-10:00 | Coffee Break | | | | 15 min |
| 10:00-10:15 | Module6  Behavior therapy technique1 | Q6 | Why would you want to lie flat? (no measure blood pressure?)  What happens if you stay flat all the time？ | Groups 1-5 discuss question 2 and Group 2 answers.  Groups 6-10 discuss question 1. Groups 6 and 8 answers. | 15 min |
| 10:15-10:40 |  | Lecture 8 | Behavioral activation | Three models: reinforcement principle, rising and falling spirals, and equilibrium model.  Arrange and make plans. | 25 min |
| 10:40-11:10 |  | Demonstration 7 | Behavioral activation | Take depression as an example. | 30 min |
| 11:10-11:40 |  | Exercise 6 | Behavioral activation | Select cases with reduced behavior and make action plans. | 30 min |
| 11:40-12:00 |  | Lecture 9 | Relaxation training | Introduce principles and common methods.  Live audio experience. | 20 min |
| 12:00-13:30 | Lunch Break | | | | 90 min |
| 13:30-13:45 | Module7  Behavior therapy technique2 | Q7 | Why do we run away?  How do you view "escape is shameful, but useful"? | Groups 1-5 discuss question 1. Groups 3 and 5 answer.  Groups 6-10 discuss question 2 and Group 7 answer. | 15 min |
| 13:45-14:15 |  | Lecture 10 | Exposure therapy | The principle and application of exposure.  Procedure and precautions. | 30 min |
| 14:15-14:45 |  | Demonstration 8 |  | Use "panic attack" as an example, discuss exposure levels. | 30 min |
| 14:45-15:15 |  | Exercise 7 |  | Select the case with "avoidance" symptoms and set the exposure level. | 30 min |
| 15:15-15:30 | Coffee Break | | | | 15 min |
| 15:30-15:45 | Module8  Psychotherapist competency development | Q8 | What does a competent psychotherapist need?  How to develop your competence? | Groups 1-5 discuss question 2 and Group 4 answer.  Groups 6-10 discuss question 1. Groups 9 and 10 answer. | 15 min |
| 15:45-16:00 |  | Lecture 11 | Competency structure of psychotherapist  The Growth path of CBT therapists | Competency components: knowledge, skills, values.  Growth path: reading, training, practice, supervision.  Introduce 8-week follow-ups training period. | 15 min |
| 16:00-17:00 | Feedback and summary | Feedback | Feelings on teaching mode and content | Briefly introduce several teaching modes used in the course, and students’ feedback. | 60 min |
| 17:00-17:15 |  | Post-test | questionnaire survey | Complete the evaluation on the effectiveness of teaching. | 15 min |

Note for learning methods: 1) Question 1-8 will use problem-based learning (PBL) and team-based learning (TBL); 2) Lecture 1-11: lecture--based learning (LBL); 3) Teachers’ presentation 1-8: Case-based learning (CBL) and experiential learning (Role-play learning); 4) Exercise: 1-7 experiential learning (Role-play learning)

DTR: Dysfunctional Thought Record; CBT: cognitive behavioral therapy; GAD: generalized anxiety disorder; SRC: stimulus-response compatibility
